# Teenage parents and their children—position paper of the European academy of paediatrics and the European confederation of primary care paediatricians

**DOI:** 10.3389/fped.2024.1418552

**Published:** 2024-07-25

**Authors:** José Fontoura-Matias, Davit George Chakhunashvili, Sian Copley, Łukasz Dembiński, Agnieszka Drosdzol-Cop, Adamos Hadjipanayis, Laura Reali, Artur Mazur

**Affiliations:** ^1^ Young European Academy of Paediatrics, Brussels, Belgium; ^2^ Department of Paediatrics, Centro Hospitalar Universitário São João, Porto, Portugal; ^3^ Department of Gynecology-Obstetrics and Paediatrics, Faculty of Medicine, Universidade do Porto, Porto, Portugal; ^4^ I. Tsitsishvili Children’s New Clinic, Tbilisi, Georgia; ^5^ Department of Paediatric Gastroenterology, Royal Manchester Children's Hospital, Manchester, United Kingdom; ^6^ Department of Pediatric Gastroenterology and Nutrition, Medical University of Warsaw, Warsaw, Poland; ^7^ European Academy of Pediatrics, Brussels, Belgium; ^8^ Department of Gynecology, Obstetrics and Gynecological Oncology, Medical University of Silesia, Katowice, Poland; ^9^ Medical School, European University Cyprus, Nicosia, Cyprus; ^10^ European Confederation of Primary Care Paediatricians, Lyon, France; ^11^ Medical Faculty, University of Rzeszów, Rzeszów, Poland

**Keywords:** adolescents, medical care, mental health, pregnancy, family

## Abstract

**Introduction:**

Teenage parenthood presents multifaceted implications, affecting adolescent parents, their children, and extended families. Despite a decrease in teenage pregnancy rates across Europe, the phenomenon continues to present significant challenges, impacting not only the adolescent parents but also their offspring and extended families.

**Methods:**

A comprehensive literature review was conducted. Key factors influencing teenage pregnancies, including socioeconomic background, family structure, and access to sex education and contraception, were examined. This review was supplemented by expert opinions from the European Academy of Paediatrics (EAP) and the European Confederation of Primary Care Paediatricians (ECPCP).

**Results:**

The triad of mother, father, and child presents individual distinct healthcare needs and vulnerabilities, highlighting the importance of specialized support and healthcare. This paper explores the psychological, social, and educational repercussions of teenage parenthood on both parents and their children, including higher risks of postpartum depression, school dropout, and repeat pregnancies. Furthermore, it underscores the critical role that paediatricians and primary care providers play in supporting these young families.

**Discussion:**

The position paper advocates for comprehensive care for adolescent parents and their children. It recommends preventive measures such as proper sex education and access to contraception to reduce unplanned teenage pregnancies. Additionally, it emphasizes the need for specialized healthcare and support for teenage parents to address their unique challenges and improve outcomes for both parents and their children.

## Introduction

Adolescence is a critical phase between childhood and adulthood that is fundamental for human development and involves physical, cognitive and psychosocial growth. Pregnancy during adolescence can have a significant impact not only on the mother but also on the father, child and grandparents. Over the last few years, rates of pregnancy in adolescence have been declining in high- and middle-income countries, with a tendency towards more effective contraception. In Europe, the lowest teen pregnancy rate was reported in Switzerland (8/1,000 adolescents), followed by the Netherlands (14/1,000), with Eastern Europe presenting a higher average prevalence (1–4). Despite this encouraging trend, adolescent pregnancy continues to be an issue that medical professionals must acknowledge. Only an extremely low percentage (1.5%) of these pregnancies were reported to have been planned (1, 5, 6). Data from the European Union shows a lack of specialised adolescent centres in nearly half of the countries and free oral contraception in only ten countries. A minority of countries have set up special programmes for pregnant teenagers and, in most of them, adolescents are only allowed to have an abortion with their parents’ consent (7).

The European Academy of Paediatrics (EAP) and the European Confederation of Primary Care Paediatricians (ECPCP) believe that it is essential to prevent unplanned teenage pregnancies through proper sex education and access to contraception (8). However, adolescent parents are still teenagers and, together with their child, should receive special healthcare and support, in which the paediatrician may play a key role. For this reason, a group of EAP and ECPCP experts prepared this descriptive review of the issue based on available research, international guidelines and clinical experience, and make suggestions for international and national states, health and education authorities, NGOs and healthcare providers.

## Adolescent mothers

### Factors associated with adolescent pregnancy and childbearing

Data from European countries show that the following factors influence adolescent pregnancy: age of first sexual intercourse; religious beliefs; disrupted family structure in childhood; socioeconomic disadvantage/place of residency; and maternal pregnancy before age 20. Also, the associations between conception in adolescence and immigrant populations, and conception in adolescence and lower educational levels (9–13). The risk of unplanned pregnancy also increased in those who did not use a safe contraceptive method in their first sexual intercourse, those who referred to difficulties with contraception compliance, and those who attended medical appointments irregularly (12, 14). When compared with adult mothers, adolescent mothers were more likely to be single, early sexual initiators, smokers, and alcohol users and presented higher sexually transmitted infection rates (15–17). They were also more likely to be underweight and less likely to have pregestational and gestational diabetes (16, 18).

### Adjustments to motherhood

Despite the challenges inherent to both adolescence and early parenting, motherhood brings a sense of meaning, maturity and responsibility to most adolescent mothers, with teenage mothers during the postpartum period expressing positive feelings more frequently than negative feelings towards pregnancy and childbirth. Sociodemographic characteristics determining teenage mothers’ attitudes towards pregnancy and childbirth included their age, marital status, current occupation, main source of income, and social support (19, 20). Affection expressed towards the child and recognition of the child's needs and interests are reduced in mothers experiencing tremendous prenatal stress and pregnancy-related social and economic concerns, representing some of the earliest modifiable risk factors for the child's life (21).

However, data show a statistically significant association between adolescent pregnancy and poor long-term mental health, with young mothers reporting a history of depression, anxiety, drug or alcohol abuse, and eating disorders (22, 23). In the postpartum period, adapting to rapidly changing bodies, pressure to return to pre-pregnancy body size, and adaptation to a new routine are all sources of stress (24). Compared with adult mothers, adolescent mothers presented significantly more depressive symptoms during pregnancy and 2–3 months postpartum. [39] Postpartum depression is a major public health problem affecting up to 57% of adolescent mothers, with a known negative impact on the child. Family conflict, less social support and low self-esteem are all associated with increased rates of depressive symptoms during the first postpartum year in teenage mothers (25). However, psychological and psychosocial interventions (such as home-visit interventions and prenatal, antenatal and postnatal educational programs) successfully reduced rates of postpartum depression symptoms (26).

According to international standards, screening for depression is recommended in the first and third trimesters of pregnancy, as well as in the postpartum period. The Edinburgh Postnatal Depression Scale questionnaire serves as a first-line diagnostic tool. Creating a support network for adolescent mothers is one of the most important preventive strategies, as it reduces the risk of postpartum depression fivefold. It is recommended that pregnant adolescent women are monitored for depression in the first and third trimesters of pregnancy, and adolescent mothers are monitored for up to a year after delivery (27).

### Breastfeeding

Initiation of breastfeeding after birth in adolescent mothers varied between 44% and 71%, which is significantly lower than in adult mothers (28–33). Moreover, these rates drop abruptly during the first months of life to 22% at six weeks and the average breastfeeding duration is only five weeks (28, 32). Data from the United Kingdom (31) showed that adolescent mothers often feel disempowered and vulnerable in the postpartum period and find it challenging to ask health professionals for help regarding breastfeeding, so bottle feeding becomes an easier and self-manageable option (34).

The most common factors associated with not breastfeeding and abandonment of breastfeeding are socioeconomic factors, planning to return to school or work, wanting to be able to leave the baby with others and inconvenience (28). Other causes include being unmarried, negative health behaviours such as smoking, and lack of prenatal care (30). Adolescent mothers also presented poorer knowledge about breastfeeding than adults, believing that formula is as good or even better than breastfeeding (28, 35). For those breastfeeding for less than one month, the most common reasons presented were having trouble with latching, perception of not having enough milk and pain associated with breastfeeding—problems that can potentially be resolved with proper lactation counselling (28). Both the WHO and UNICEF recommend exclusive breastfeeding during the first six months. Young breastfeeding mothers may additionally benefit from increased intervals between pregnancies and stronger mother-infant bonding (27).

### Education perspectives

There is a solid cause-and-effect relationship between teenage pregnancy and school dropout, which is reinforced by economic vulnerability (36, 37). In multiparous teenage mothers, this trend is even worse, with a mean drop out of school at 13 years, with only 10% of adolescents still attending school when they were interviewed in the postpartum period (38). In the long term, tertiary education completion was lower in the adolescent mothers’ group, suggesting that education opportunities remain compromised for those who became parents during adolescence (39). Adolescent mothers have worse outcomes in terms of education, work and training than those who were never pregnant, so it is vital to enable these mothers to return to school and gain formal education, which will increase their chances of self-sufficiency and economic stability.

### Repeated pregnancies

The birth of a second child in adolescence has a negative impact, worsening the outcomes in the different spheres of the mother's and child's lives. Adolescents with repeat pregnancies were more likely to drop out of school after their first pregnancy to focus on parenting and had a more passive attitude towards contraception. In contrast, adolescents with no repeat pregnancies actively sought long-term contraceptive methods, motivated mainly by the desire to return to studies (40). Higher levels of education and school continuation were found to be protective, while depression, history of abortion and relationship factors, such as lack of partner support, increased the risk of repeated pregnancy (41). Compared with those who were pregnant for the first time, young women who had a previous pregnancy and who terminated a pregnancy were more likely to be single and report greater substance use (smoking and alcohol dependence), and those who had a miscarriage more commonly reported depressive symptoms, binge drinking, and dependence on nicotine and cannabis (39). Most of the adolescents with repeat pregnancies report becoming pregnant again too soon and express a strong desire for contraceptive counselling (42). Data from 250 adolescent deliveries in Sweden showed that only 71% of the mothers received contraceptive prescriptions on discharge, with 25% presenting a new pregnancy at the 12-month follow-up. Of those, 36% had a legal abortion (43).

The high efficacy of long-acting reversible contraception (LARC) depends on the adolescent initiating and maintaining the contraception. Data showed that adolescents who were evaluated less often by healthcare professionals were less likely to initiate LARC, with a significantly higher risk of repeat pregnancy (44). Provision of free-of-charge contraception and over-the-counter emergency contraception regardless of age, as well as increased education, was significantly associated with lower teenage pregnancy and induced abortion rates (45). Home visits of postnatal healthcare professionals were also associated with increased contraception knowledge (46). Furthermore, in adolescent mothers offered immediate postpartum contraceptive implants, repeat pregnancy rates were significantly lower, and this strategy was cost-effective (47).

## Adolescent fathers

There is a paucity of data on issues relating to fathers in the context of adolescent pregnancy compared to data available from the mothers’ perspective. The literature suggests that fathers tend to be slightly older than mothers in the context of adolescent pregnancy, although only a minority are significantly older (48). Demographics of adolescent fathers are similar to mothers, that is, they are more likely to be from lower socioeconomic backgrounds, experience lower educational attainment and have fewer employment opportunities. There are also higher psychosocial and behavioural difficulty rates than in adolescents without children. Moreover, health-jeopardising behaviour rates are higher in teenage males involved in a pregnancy (regardless of outcome) than those never involved (49). It is also not uncommon for the child's father to be unknown or entirely uninvolved in the care of the child.

### Adjustments to fatherhood

Again, there is a lack of literature regarding adjustment to fatherhood for adolescent fathers compared to adjustment to motherhood for adolescent mothers. Adolescent fathers often have a close relationship with the mother but struggle with the new role and lifestyle changes (50). As previously discussed, adolescent fathers are more likely than other adolescent males to have behavioural difficulties. However, there is a lack of evidence as to whether this is a cause or effect of early fatherhood (49). Nevertheless, after adjusting for lower socioeconomic class and lower educational ability, a UK review found that men who become fathers in their teens or early twenties are twice as likely to be unemployed, receive benefits and require social housing (51).

### Father involvement and its impact

Paternal involvement is variable. Some studies show a decline in paternal involvement is associated with relationship breakdowns, which are common in pairs of teenage parents (48). Fathers cite conflicting relationships with the child's mother or maternal grandparents and financial stress as barriers to involvement, and adolescent fathers express the desire for greater involvement with their children (48). However, mothers are more likely to cite a lack of paternal interest as the reason for the lack of involvement, suggesting a disconnect between adolescent mothers’ and fathers’ views. It is worrying that many young fathers report being excluded from the pregnancy by healthcare providers, which may negatively impact their future involvement after the child is born (51).

### Fatherhood interventions

There is limited literature on interventions for adolescent fathers. Interventions such as home visits by nurses are associated with higher levels of parental engagement (51). Highlighting male responsibility and dispelling negative stereotypes of adolescent fathers, delivering the intervention in the participant's household, group participation and peer involvement were highlighted in a review that mapped areas for improvement in outcomes for young fathers (52). Alongside this, education for healthcare providers and promoting an inclusive approach to the young father may promote sustained involvement of the father (51, 52).

## Children of adolescent parents

Adolescent mothers are more likely to experience ill health and complications in pregnancy (53). Children born to adolescent parents are more likely to be born preterm, at a lower birth weight and are at risk of associated neonatal complications (54, 55).

### Development

There are no significant differences in development between infants born to adolescents and older mothers, but differences emerge in the preschool and school years (53). Children of adolescent parents show delays in cognitive development and behavioural difficulties (56). Adolescents experience greater difficulty at school, higher rates of antisocial behaviour and are more likely to engage in early sexual behaviour. However, some of these studies did not take other factors, such as poverty and socioeconomic status, into account.

### Safeguarding

Children born to adolescent parents may be more likely to experience neglect or other forms of child abuse, although this requires further study to explore whether this is an association rather than a causative factor (53, 57). Children of adolescent parents are more likely to become adolescent parents themselves; reported figures suggest 25%–30% of girls and 11% of boys born to adolescent parents become adolescent parents themselves (53).

Children born to teenage parents are also more likely (40% higher) to experience accidental and non-accidental injuries (57). This effect remains even when adjusting for socioeconomic variables, parental substance misuse and psychiatric illness.

## An important role for paediatricians and general practitioners

A complete social history should be obtained by the paediatrician because an assessment of family resources and support systems is necessary to care for the child and adolescent parents (58, 59). Attention should be paid to medical insurance, housing conditions, transportation, and access to food and sources of financial support. Social determinants of health—including poverty, residential mobility, limited resources, housing conditions and low financial support—significantly impact adolescent mothers (60). Data show that adolescent pregnancy increases the risk of being removed from shelters and being a victim of violence from the partner (61). In such cases, a referral from the paediatrician to the community and social support services might be favourable. Poverty significantly affects adolescent parents and their children and is associated with repeated pregnancies (37, 62). Here, the key strategy is helping parents access job training for themselves and childcare programs for their children. Repeated adolescent pregnancies limit educational achievements and increase the risk of child neglect and parenting stress (63, 64). Lack of family support, poor education levels and poverty may also negatively impact prenatal care, which may lead to health issues for the child and adolescent mother, including insufficient nutrition, low birth weight, preterm birth, pre-eclampsia and anaemia (65). Positive health outcomes may help adolescent mothers set educational goals and positive attitudes and build strong social support networks.

During the visit with the paediatrician, meeting extended family members, who might eventually be helping with the childcare, is of great importance. In many cases, grandparents provide financial and emotional support, taking responsibility for the child's care and mentoring adolescent parents. It is also reported that maternal grandparents often view an infant as a source of firm family connections (66). An adverse family reaction to the pregnancy announcement may negatively impact the future relationship between the adolescent and her family, with adolescent females presenting higher risks for depression if they have a bad relationship with their own mothers (67–69). Although childcare assistance from family members (including grandparents) is important for the adolescent mother, the development of autonomy may be essential to adapt to her own parenting journey (69).

Society and community may treat adolescent mothers and fathers like outcasts, view them as irresponsible or be ambivalent towards their children and future career goals (70, 71). Pregnant and adolescent mothers may sometimes experience mistreatment, lack of empathy, and discriminatory attitudes even from healthcare providers (70). Despite pervasive stereotypes about adolescent mothers and fathers, adolescent parents must be fully supported, and positive aspects should always be highlighted. In adolescent mothers, lack of social support may be associated with increased rage and punitive parenting behaviour (72). Different studies show the importance and benefits of support groups in improving self-esteem, communication and parenting skills (73, 74).

A detailed history of substance use, as well as any risk of child abuse, should be obtained from the adolescent mother and father by the paediatrician during office visits (75). Identification of substance use and prompt referral to treatment is critical for all adolescents, especially pregnant teenagers (75, 76).

Screening for intimate partner violence (IPV) among adolescent parents prepartum and after pregnancy is essential. IPV includes psychological aggression, physical and sexual violence, stalking, verbal abuse and sexual and reproductive health control (69). Motherhood and adolescent pregnancy are generally associated with an increased risk of sexual, physical and emotional violence (70). Adolescent mothers’ views about IPV may be normalised or complicated if they previously experienced violence as a child (77). In some cases, adolescent females may not report abuse and stay with the violent father to maintain their partner in the child's life (78). Lower levels of interparental conflict and access to family resources may promote an enhancement in the developmental gains of the child in the postnatal period (75). In order to assess IPV, paediatricians can use effective self-administered surveys like HITS (Hurt, insulted, threatened and screamed) and WATS (Woman abuse screening tool) (79, 80).

**Based on available data and clinical experience, the EAP and ECPC expert group recommends:**

For international and national states, health and education authorities and NGOs:

• Pregnancy in adolescents’ issue should be part of undergraduate and postgraduate training for healthcare providers.
• Creating adequate shelters for teenage parents in a difficult life situation should be supported.
• Social campaigns against the stigmatisation of adolescent pregnancy should be organised.
• Multidisciplinary care should be easily accessible to the adolescent early in the pregnancy.

For healthcare providers:

• The role of healthcare providers is to inform adolescents about effective methods of preventing unwanted pregnancy and sexually transmitted diseases.
• In the case of teenage pregnancies, healthcare providers should encourage and facilitate adolescents to receive appropriate gynaecological and obstetric care, e.g., by indicating where such services are provided free of charge.
• In the case of pregnant adolescents, increased vigilance regarding possible violence and the use of drugs is recommended.
• Teenage parents and their children require special support and information on childcare and prevention, including methods of care, calming, feeding, supporting development and vaccinations for the child.

## Data Availability

The original contributions presented in the study are included in the article/Supplementary Material, further inquiries can be directed to the corresponding author.
